# Dysplastic Hips in Young Adults Demonstrate Different Relationships Between Acetabular Coverage, Joint Congruity, and Contact Mechanics Than Asymptomatic Hips

**DOI:** 10.1002/jor.70048

**Published:** 2025-08-25

**Authors:** Holly D. Aitken, Jessica E. Goetz, Wyatt M. Sailer, Dominic J. L. Rivas, Krit Petrachaianan, Natalie A. Glass, Michael C. Willey, Joshua B. Holt

**Affiliations:** ^1^ Department of Orthopedics & Rehabilitation University of Iowa Iowa City Iowa USA; ^2^ Department of Biomedical Engineering University of Iowa Iowa City Iowa USA

**Keywords:** acetabular coverage, contact stress, discrete element analysis, hip dysplasia, joint congruity

## Abstract

This study investigated the relationship between three‐dimensional (3D) acetabular coverage and contact mechanics in dysplastic and ostensibly normal hips. Fifty asymptomatic hips previously imaged with CT scans/angiograms were matched on a 2:1 basis to 25 dysplastic hips with previous CT imaging, based on age, gender, weight, and BMI. CT imaging was used to create 3D patient‐specific hip models from which the 3D coverage metrics of subchondral arc angle (i.e., acetabular weight‐bearing morphology) and hip joint coverage angle (i.e., femoral head coverage), and the congruity metrics of acetabular sphericity index SI (i.e., sphericity of the acetabulum) and joint congruity index CI were assessed globally and in five octants spanning the weight‐bearing acetabulum. Discrete element analysis was used to calculate hip contact mechanics, with results assessed globally and subdivided into the same five octants. Increasing superior‐anterior subchondral arc angle was associated with *increasing* superior‐anterior mean chronic contact stress‐time exposure in dysplastic hips, which was significantly (*p* < 0.001) different from asymptomatic hips where increasing superior‐anterior subchondral arc angle was associated with *decreasing* superior‐anterior mean chronic contact stress‐time exposure. Similarly, increasing joint congruity CI anteriorly was associated with *increasing* anterior mean chronic contact stress‐time exposure in dysplastic hips, which was significantly (*p* = 0.003) different from the trend of *decreasing* anterior mean exposure with increasing anterior CI in asymptomatic hips. These results indicate fundamental differences in how contact mechanics in asymptomatic and dysplastic hips respond to differences in acetabular coverage and joint congruity, suggesting that asymptomatic hips follow the expected geometry‐based trend, while dysplastic hips do not.

## 1 Introduction

Acetabular development is a highly complex process that involves interstitial growth in the triradiate acetabular cartilage, appositional growth along the triradiate cartilage periphery, and periosteal ossification at the acetabular margin, followed by ossification in the hyaline cartilage around the acetabular cavity from secondary centers of the pubis, ilium, and ischium [1]. Alterations in the acetabular growth process result in malformation of the lunate surface, referred to as acetabular dysplasia. Hip dysplasia describes a complex spectrum of acetabular and femoral deformity in which a shallow acetabulum provides insufficient femoral head coverage, creating an incongruous, unstable hip joint [2]. The most common surgical treatment for hip dysplasia after skeletal maturity is the periacetabular osteotomy (PAO), which involves multiplanar acetabular realignment with the goals of correcting coverage deficiencies, stabilizing the hip joint, and medializing the joint center [3]. Given the invasive nature of this surgical procedure, there has been an increased focus on determining specific structural deformities of a dysplastic hip that need to be corrected with a PAO to improve patient symptomatology and reduce the risk of premature osteoarthritis [4, 5, 6].

While clinical assessment of hip dysplasia severity is typically based on two‐dimensional (2D) radiographic measurements [7], these measures can have highly variable interrater reliability (0.38–0.94) [8, 9] that is influenced by the radiographic view acquired, measurement technique used, and pelvis orientation during radiograph acquisition [10]. To address limitations of 2D radiographic measurements in fully assessing complex, three‐dimensional (3D) joint morphology, several methods for quantifying 3D acetabular coverage have been developed [11, 12, 13, 14, 15, 16, 17, 18, 19, 20, 21, 22, 23]. We have previously developed a custom, semi‐automated algorithm that evaluates regional acetabular coverage using 3D measures that nominally relate to traditional 2D radiographic coverage measures [24].

In a nominally spherical articulation like the hip joint, increasing acetabular coverage of the femoral head is assumed to increase load‐bearing surface area [25] and reduce contact stress [5, 6]. Consequently, reduced contact areas and associated contact stress elevations in dysplastic hips [26, 27] could be returned to normal levels through surgical correction with PAO. However, prior studies have demonstrated that even when femoral head coverage is increased to radiographically normal levels via PAO, the expected improvements in contact mechanics are not always seen [5, 6, 26, 28]. It is unclear why a disconnect exists between how a ball‐and‐socket joint is expected to behave mechanically and how contact mechanics in dysplastic hips change in response to acetabular coverage alterations. Defining how 3D acetabular coverage relates to hip contact mechanics may provide surgeons information on how to better reduce abnormal contact stresses without generating a full patient‐specific computational model.

The objective of this study was to investigate the relationship between 3D acetabular coverage and contact mechanics in both dysplastic and ostensibly normal asymptomatic hips. We hypothesized that both dysplastic and asymptomatic hip joints would demonstrate strong relationships between 3D acetabular coverage and contact mechanics.

## 2 Methods

### 2.1 Patients Modeled

Previously, a cohort of 100 subjects (125 hips) with hip dysplasia treated with PAO at our institution between December 2002 and July 2010 was identified that had preoperative and postoperative pelvic CT scans available for patient‐specific discrete element analysis (DEA) [29]. From this cohort, 21 subjects (25 hips) between the ages of 17 and 25 years were selected for inclusion in the current study.

With Institutional Review Board approval, hips of 50 asymptomatic subjects were identified based on current procedural terminology codes as having undergone pelvis/hip CT imaging or abdomen/pelvis CT angiograms at our institution between January 2018 and December 2021 [24]. CT imaging indications included unilateral pelvic/femur fracture, kidney donor candidacy evaluation, abdominal cyst and/or abscess, hematoma/hemorrhage, arterial/vascular disorders, and standard workup following motor vehicle collision. These 50 subjects were matched on a 2:1 basis to the 25 dysplastic hips based on age, gender, weight, and BMI to increase statistical power. Beyond matching criteria, inclusion criteria included no history of congenital or developmental hip pathology, no diagnosed neuromuscular conditions, and no bilateral pelvic and/or proximal femoral fractures. A random hip side was selected for analysis in subjects without pelvic or proximal femoral fractures; for subjects with a unilateral fracture, the nonfractured side was selected. Comparisons of subject demographics and radiographic evaluation between asymptomatic and dysplastic hips are shown in Tables 1 and 2.

**Table 1:** Comparison of subject demographics between asymptomatic and dysplastic hips. Data are presented as median [IQR] except for gender, which is presented as number of females [%].

	Age (years)	Weight (kg)	BMI (kg/m^2^)	Height (m)	Gender (# of females [%])
Asymptomatic (*n* = 50 hips in 50 subjects)	21 [19–23]	66.5 [57.5–76.3]	22.5 [21.4–26.9]	1.7 [1.6–1.7]	41/50 [82%]
Dysplastic (*n* = 25 hips in 21 subjects)	19 [18–22]	71.0 [58.5–75.9]	22.5 [21.1–25.6]	1.7 [1.6–1.7]	19/21 [90%]
*p*‐value	0.145	0.948	0.973	0.673	0.488

**Table 2:** Comparison of radiographic evaluation between asymptomatic and dysplastic hips. Data are presented as median [IQR].

	Lateral center edge angle (°)	Acetabular arc angle (°)
Asymptomatic (*n* = 50 hips in 50 subjects)	29 [25–34]	61 [56–68]
Dysplastic (*n* = 25 hips in 21 subjects)	17 [8–20]	61 [56–64]
*p*‐value	**< 0.001**	0.318

*Note:* Bolded text indicates statistical significance.

### 2.2 Model Generation

Each subject's pelvic and proximal femoral geometry was segmented using either a watershed‐based MATLAB (Mathworks, Natick, MA, USA) algorithm with manual correction of edge definition errors or holes in cancellous bone, or using a threshold‐based segmentation performed in MIMICS image processing software (Materialise, Leuven, Belgium) with similar manual corrections. The minimal variability in contact stress calculations due to the different segmentation protocols has been previously reported [29].

Segmented scans were converted to 3D triangulated surface models and smoothed to eliminate stair‐step artifact [28]. Regions of interest (ROIs) on the pelvis and femur were manually identified from the smoothed surface models. The acetabular ROI included all pelvic surface area between the lateral acetabular rim and medial rim separating the acetabular fossa from the acetabular lunate. The femoral head ROI encompassed all femoral head surface area, including the fovea and down to the rim delineating the convex femoral head from the concave femoral neck. In femurs with cam deformity in which flattened regions obscured this convex/concave transition, the “rim” that would have existed if the cam deformity was not present was used to define the femoral head ROI.

### 2.3 Articular Cartilage Approximation

To approximate articular cartilage from the bone surface models, the acetabular and femoral head ROI surfaces were projected into the joint a uniform distance equal to half of the mean joint space width, and these projections were then smoothed using a combination of radial and nearest‐neighbor techniques [30] to generate continuous, nonspherical, and nonuniform‐thickness cartilage surfaces. Models generated using this methodology have been shown to produce contact stress calculations that have good agreement with experimentally measured pressures in identically loaded cadaveric hip joints [30].

### 2.4 Standardized, Neutral Model Orientation

Each hip model (pelvis/femur bone surfaces, acetabular/femoral head ROI surfaces, and acetabular/femoral head cartilage surfaces) was oriented to a standardized and neutral, anatomic landmark‐based hip joint coordinate system [31]. The pelvis orientation was prescribed based on the locations of the left and right anterior superior iliac spines and the midpoint of the left and right posterior superior iliac spines and with the origin located at the center of a best‐fit sphere to the femoral head ROI. As the patient‐specific imaging used in this study did not include the distal femur, orienting the patient‐specific femur models was achieved by aligning each to a full‐length, template femur model with nominally normal (9°) femoral version that was already oriented in the coordinate system based on the location of its femoral head center and the midpoint of the medial and lateral femoral epicondyles [32]. After surface alignment, each patient‐specific femur was translated to place the center of the best‐fit sphere to the patient‐specific femoral head ROI at the origin, rather than at the femoral head center of the template femur. This allowed the pelvis and femur models to be rotated to a prescribed neutral orientation while maintaining the patient‐specific acetabular‐femoral positional relationship.

### 2.5 3D Acetabular Coverage Assessment

**Figure 1:**
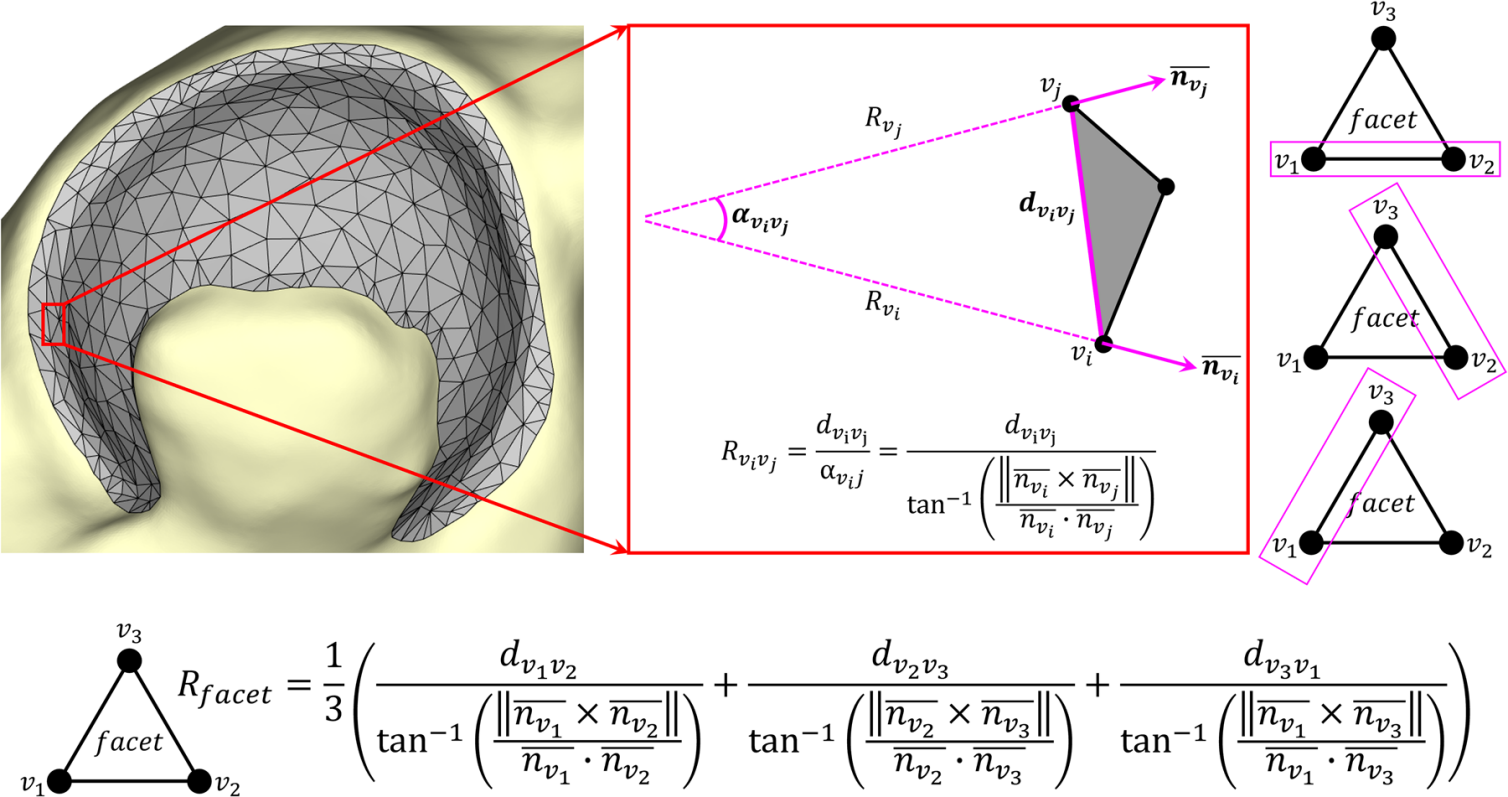
Calculation of the radius of curvature for each triangular facet (gray), based on the cross and dot products of the surface normals of triangular vertices [24]. For each triangular vertex v (black dot) of a given triangular facet, the surface normal $\bar{n_{v}}$ (pink arrow) was approximated by averaging the surface normals of all triangular facets attached to vertex v. For each pair of vertices $v_{i}$ and $v_{j}$ of a given triangular facet (i.e., one side of the triangle, pink boxes), the Euclidean distance between those vertices ($d_{v_{i}v_{j}}$) and the angle between the surface normals of those vertices ($\alpha_{v_{i}v_{j}}$, computed using the cross product and dot product of the surface normals of vertices $v_{i}$ and $v_{j}$) were calculated. The ratio of $d_{v_{i}v_{j}}$ and $\alpha_{v_{i}v_{j}}$ provided the approximation of the radius of curvature $R_{v_{i}v_{j}}$ for that triangular side. The curvature radii for all three sides were averaged to obtain a single radius of curvature $R_{\mathrm{facet}}$ value for each triangular facet.

**Figure 2:**
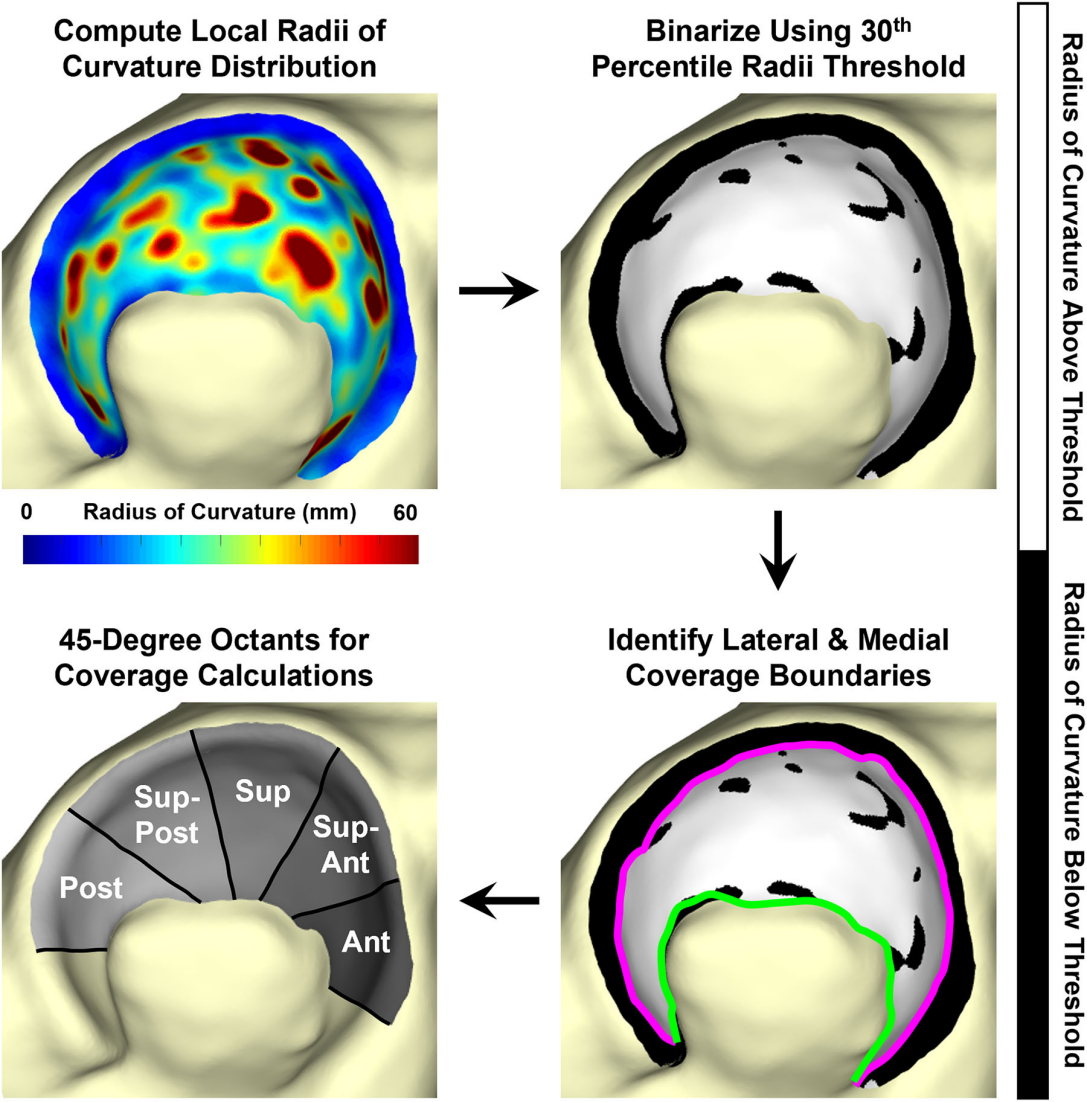
Process of identifying the lateral and medial‐most boundaries of the acetabular rim using the curvature radii distribution [24]. A custom MATLAB algorithm computed the localized radius of curvature distribution, and this distribution was then smoothed using a nearest‐neighbor algorithm (top left panel). The smoothed distribution was then binarized using a 30th percentile radius of curvature threshold to select concave locations (top right panel), and all triangular facets with curvature radii values greater than the threshold (white) were isolated from the remainder of the acetabular region of interest (ROI). A boundary was automatically identified around the centroids of these concave triangular facets and then manually separated into lateral (pink) and medial (green) boundaries (bottom right panel). The lateral and medial boundaries were used for assessing 3D acetabular coverage in each of five octants comprising the weight‐bearing portion of the acetabular ROI (bottom left panel).

**Figure 3:**
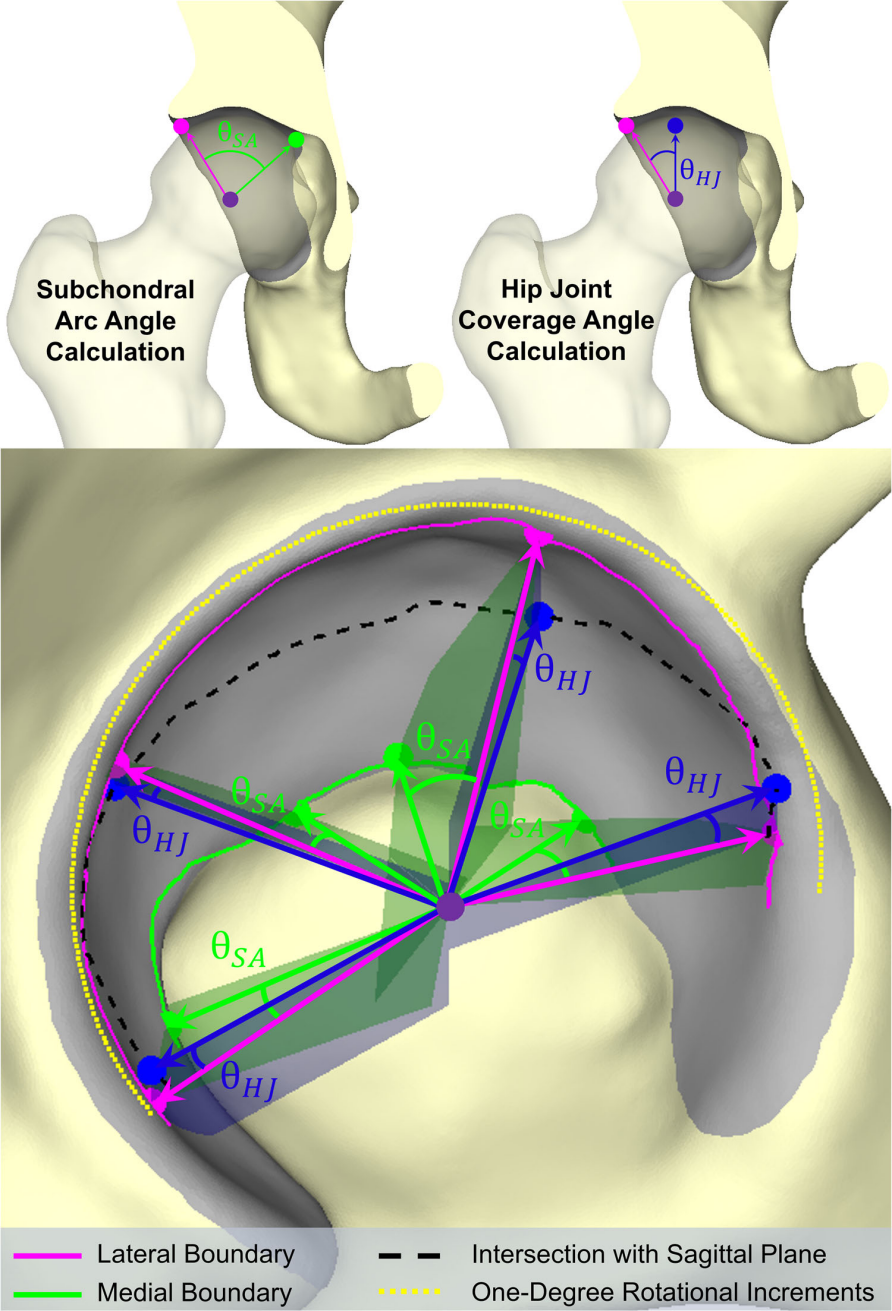
(Top left) The 3D subchondral arc angle, $\theta_{\mathrm{SA}}$, describes the weight‐bearing acetabular anatomy and was defined as the angle between (1) a vector from the center of the femoral head (purple dot) to the triangular facet that was closest to the lateral acetabular boundary (pink dot) and (2) a vector from the femoral head center (purple dot) to the triangular facet that was closest to the medial acetabular boundary (green dot). (Top right) The hip joint coverage angle, $\theta_{\mathrm{HJ}}$, describes femoral head coverage provided by the acetabulum and was defined as the angle between (1) a vector from the femoral head center (purple dot) to the lateral acetabular boundary (pink dot) and (2) a vector oriented in the same anterior‐posterior and superior‐inferior directions as (1) but without a medial‐lateral component (blue dot). $\theta_{\mathrm{HJ}}$ can also be described as the angle between a vector from the femoral head center to the lateral boundary and a sagittal plane located at the femoral head center. (Bottom) Examples of both $\theta_{\mathrm{SA}}$ (green angles) and $\theta_{\mathrm{HJ}}$ (blue angles) computed at multiple rotational increments around the acetabular circumference. The green planes demonstrate the planes in which $\theta_{\mathrm{SA}}$ is being calculated at varying angles around the acetabulum. Likewise, the blue planes indicate the planes in which $\theta_{\mathrm{HJ}}$ is being calculated at the same angles around the acetabulum. While only four examples of $\theta_{\mathrm{SA}}$ and $\theta_{\mathrm{HJ}}$ are shown, both angles were computed in 1° rotational increments around the acetabular circumference (yellow dots) using the entire lateral acetabular boundary (pink line), medial acetabular boundary (green line), and locations on the acetabulum intersected by a sagittal plane located at the femoral head center (black dashed line). Once $\theta_{\mathrm{SA}}$ and $\theta_{\mathrm{HJ}}$ were computed at every 1° rotational increment around the acetabular circumference, angles were averaged globally and within each of the five octants comprising the weight‐bearing portion of the acetabular ROI [24]. $\theta_{\mathrm{HJ}}$ was computed for each of the 13 gait cycle orientations separately, and the average global and regional $\theta_{\mathrm{HJ}}$ values for those 13 orientations were computed.

A previously developed MATLAB algorithm [24] was used to calculate the local radius of curvature of each triangular facet on the acetabular ROI (Figure 1), based on the cross and dot products of the triangular vertex surface normals. The algorithm then semi‐automatically identified the medial and lateral boundaries of the weight‐bearing acetabulum (Figure 2). These boundaries were used to compute (1) the 3D subchondral arc angle, $\theta_{\mathrm{SA}}$, which is a static measure describing weight‐bearing acetabular morphology, and (2) the 3D hip joint coverage angle, $\theta_{\mathrm{HJ}}$, which more closely describes functional acetabular coverage of the femoral head and changes with hip position during stance phase of gait (Figure 3) [24]. Both $\theta_{\mathrm{SA}}$ and $\theta_{\mathrm{HJ}}$ were computed in 1° rotational increments around the acetabular circumference (Figure 3). Individual values were averaged within each of five, 45° octants comprising the weight‐bearing portion of the acetabular ROI (Figure 2) [24] and across the entire weight‐bearing acetabulum (global).

### 2.6 Discrete Element Analysis‐Computed Contact Mechanics

Each hip model was loaded with 13 independent sets of hip joint reaction forces and rotation angles spanning stance phase of walking gait. These were obtained by discretizing average continuous hip joint loading patterns reported in the literature. For the dysplastic hip models, the average loading pattern was derived from 10 patients with hip dysplasia [33]. For the asymptomatic hip models, the average loading pattern was obtained from 10 control subjects with no history of hip dysfunction and no evidence of dysplasia or OA, and who were similar in age, weight, height, and BMI to the patients with dysplasia [33]. The discretized hip joint reaction forces were scaled according to the bodyweight of the individual being modeled. Cartilage was assigned isotropic, linear‐elastic material properties (*E* = 8 MPa, *ν* = 0.42) [28, 34]. DEA calculations were performed using a Newton's method solver implemented in MATLAB [35].

Contact stresses calculated for each quasi‐static loading configuration were multiplied by the length of time spent in the given configuration and summed across all 13 time points to obtain the cumulative contact stress‐time distribution per single walking step (in MPa‐s) [35]. This per‐step data was multiplied by an assumed 2 million walking steps per year [36, 37] and the subject's age in years to obtain the chronic contact stress‐time exposure distribution (in MPa‐years) [30]. In each model, the peak and mean contact stress, peak and mean chronic contact stress‐time exposure, mean contact area (areas experiencing contact averaged across all 13 gait step increments), and cumulative contact area (total area that experiences contact during any of the 13 gait step increments) during the gait cycle were computed for the entire weight‐bearing acetabulum (global) and each octant of interest.

### 2.7 Joint Congruity

**Figure 4:**
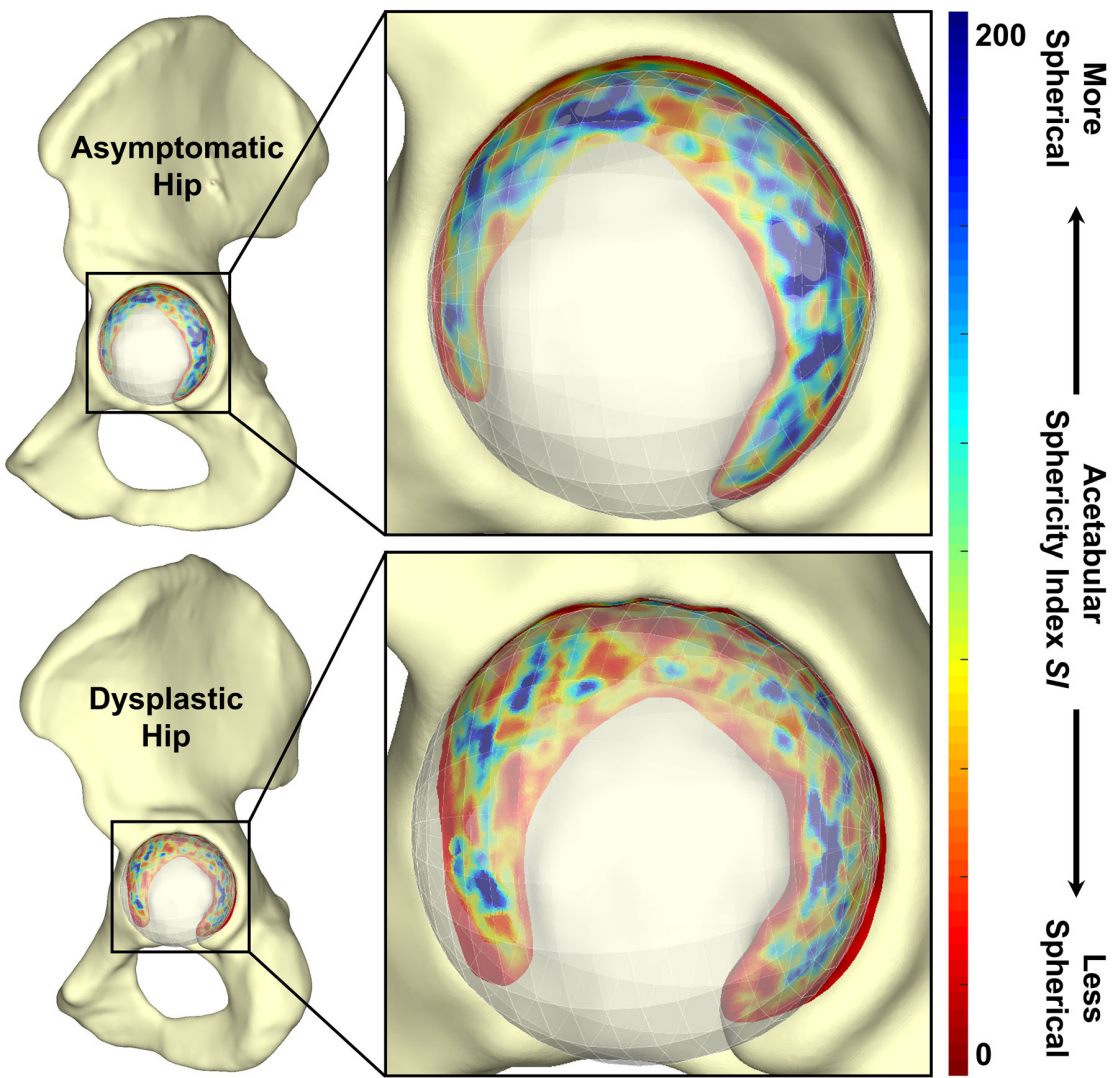
Example acetabular sphericity index (SI) distributions for a dysplastic hip and one of the asymptomatic hips matched to it. Smaller acetabular sphericity index values (red) indicate a less spherical acetabulum, whereas higher values (blue) indicate a more spherical shape of the acetabulum. The increased amount of blue in the acetabular sphericity index distribution for the asymptomatic hip indicates a more spherical acetabulum than in the corresponding dysplastic hip.

While contact stress is dependent on coverage of the femoral head by the acetabulum, it also depends on the congruency between the acetabular and femoral head articular surfaces. To parallel the calculations that were performed for coverage, both an acetabular “congruity” metric and a joint congruity metric (incorporating acetabular and femoral interaction) were calculated. To describe acetabular morphology, a congruity index [38] was calculated between the acetabular ROI and an idealized sphere fit to the acetabular ROI. This index, the acetabular sphericity index (SI, Figure 4), was computed for each acetabular triangular facet t on the acetabular ROI as

$${\mathrm{SI}}_{t}=\frac{1}{\left\|\frac{1}{r_{c_{a}}}n_{s_{a}}-\frac{1}{r_{c_{s}}}n_{s_{s}}\right\|},$$

where $r_{c_{a}}$ is the radius of curvature of the acetabular triangular facet t, $n_{s_{a}}$ is the surface normal of acetabular triangular facet t, $r_{c_{s}}$ is the radius of an idealized sphere fit to the acetabular ROI, and $n_{s_{s}}$ is the normalized vector from the center of the idealized sphere fit to the acetabular ROI to the centroid of acetabular triangular facet t. Individual facet SI values were averaged within each of the five octants of interest and across the entire weight‐bearing acetabulum (global).

Similarly, joint congruity was calculated between the acetabular and femoral head ROIs at each of the 13 gait step increments (i). To determine which acetabular and femoral head facets were in contact at each gait increment, the femoral head was rotated to the specified gait orientation, and for each acetabular triangular facet, a ray was cast along the direction of the surface normal until it intersected a triangular facet on the femoral head ROI. Only pairs of acetabular and femoral head triangular facets that were within 5 mm of each other were included in the joint congruity calculation. The joint congruity index (CI) [38] was computed for each acetabular triangular facet t by modifying the SI calculation such that $r_{c_{s}}$ is the radius of curvature of the femoral head triangular facet associated with acetabular triangular facet t, and $n_{s_{s}}$ is the surface normal of the femoral head triangular facet associated with acetabular triangular facet t. The mean CI was computed for each gait step increment and then averaged across all 13 gait steps to obtain the mean CI during gait for the entire weight‐bearing acetabulum (global) and each octant of interest.

### 2.8 Statistical Analysis

Differences in global and regional 3D acetabular coverage metrics, joint congruity, and contact mechanics metrics were compared between asymptomatic and dysplastic hips using Wilcoxon rank sum tests with Holm‐Bonferroni corrections for multiple comparisons across acetabular regions performed in SAS 9.4 (SAS Institute Inc, Cary, NC, USA). Mixed‐effect linear models with random intercepts were used to assess the effects of (1) weight‐bearing acetabular morphology (subchondral arc angle [$\theta_{\mathrm{SA}}$]) and (2) acetabular sphericity (SI) on contact mechanics metrics, with acetabular region and diagnosis of hip dysplasia as covariates. Similarly, another set of mixed‐effect linear models was used to assess the effects of (1) joint coverage (hip joint coverage angle ([$\theta_{\mathrm{HJ}}$]) and (2) joint congruity (CI) on contact mechanics, with the same covariates of acetabular region and diagnosis of hip dysplasia. Analyses using mixed‐effect models were performed in R v4.1.0 (R Core Team, Vienna, Austria) using the *nlme* package [39].

## 3 Results

### 3.1 Global and Regional Differences Between Asymptomatic and Dysplastic Hips

**Figure 5:**
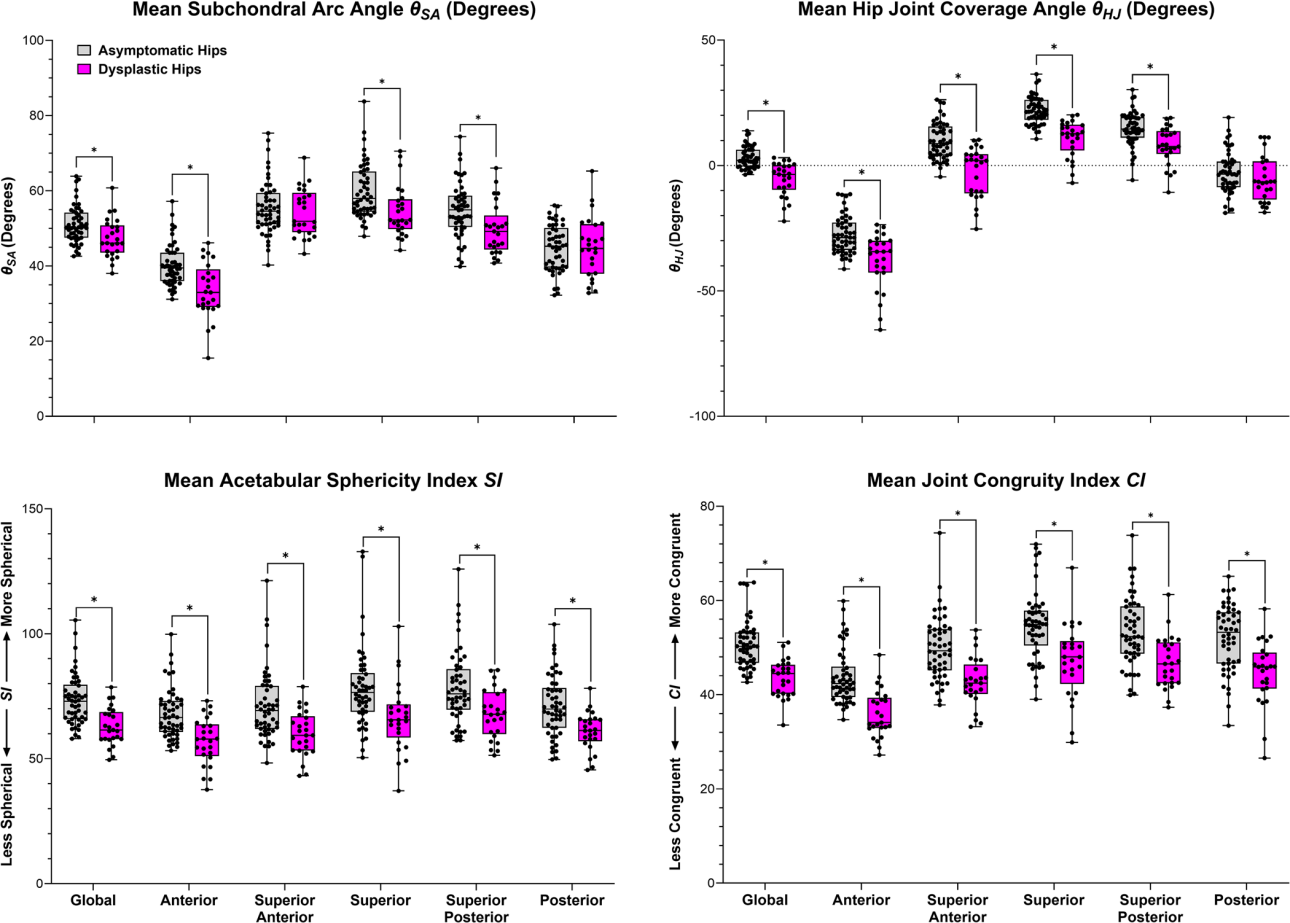
Boxplots illustrating regional differences in 3D acetabular coverage and joint congruity metrics between asymptomatic (gray) and dysplastic (pink) hips. *p < 0.05 after adjustment for multiple comparisons.

Dysplastic hips had significantly less global, anterior, superior, and superior‐posterior coverage than asymptomatic hips, both in terms of weight‐bearing acetabular morphology (subchondral arc angle [$\theta_{\mathrm{SA}}$], *p* ≤ 0.012) and coverage of the femoral head (hip joint coverage angle [$\theta_{\mathrm{HJ}}$], *p* < 0.001) (Figure 5). Globally, and in all weight‐bearing regions, dysplastic hips had significantly less spherical acetabula (acetabular sphericity index [SI], *p* ≤ 0.002) and less congruent hip joints (joint congruity index [CI], *p* < 0.001) than asymptomatic hips (Figure 5).

**Figure 6:**
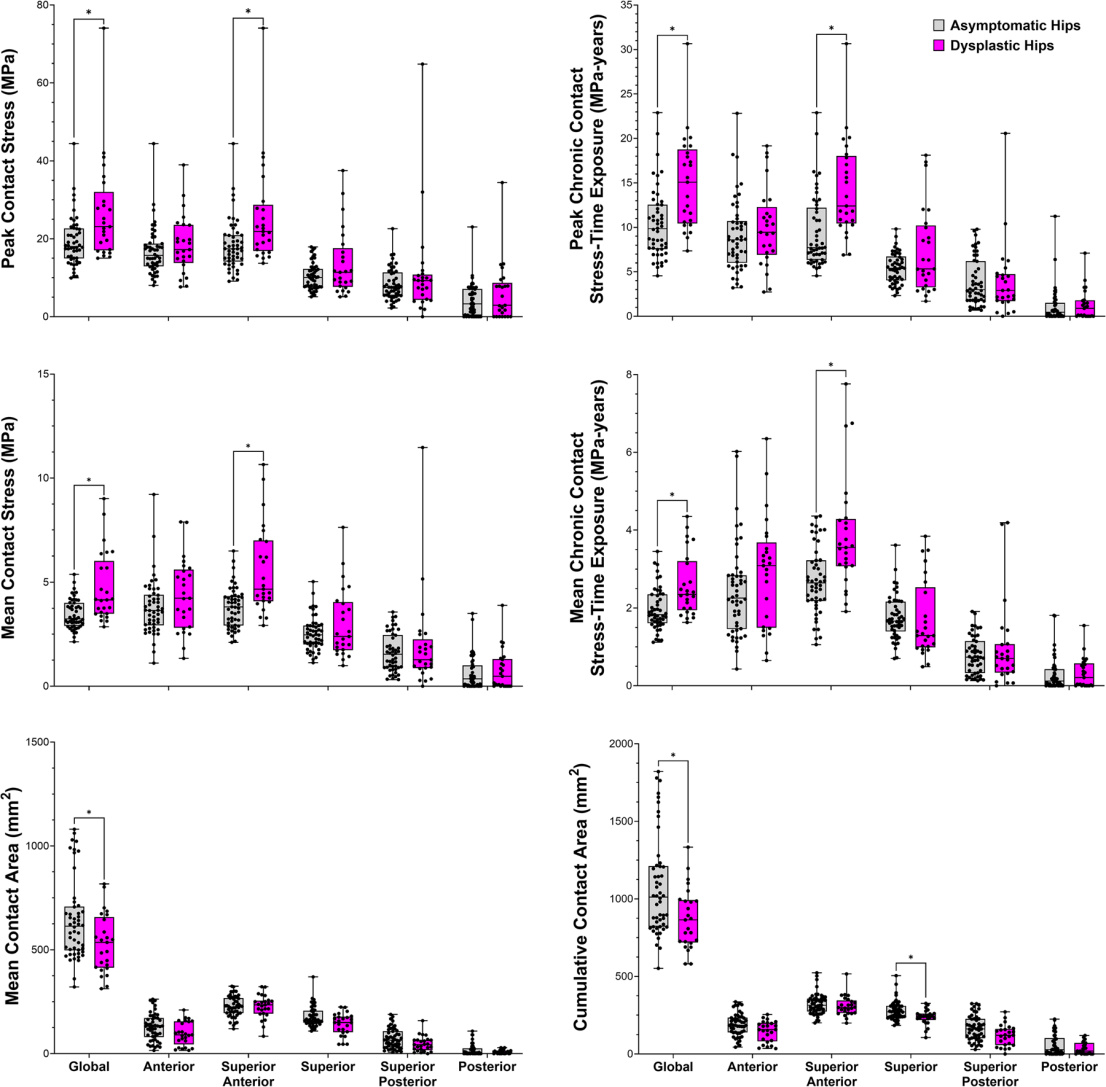
Boxplots illustrating regional differences in contact mechanics metrics between asymptomatic (gray) and dysplastic (pink) hips. *p < 0.05 after adjustment for multiple comparisons.

Globally, asymptomatic hips had significantly greater mean (*p* = 0.018) and cumulative (*p* = 0.005) contact area than dysplastic hips (Figure 6). Asymptomatic hips also had significantly greater cumulative contact area in the superior region (*p* = 0.031) than dysplastic hips (Figure 6). Dysplastic hips demonstrated significantly (*p* ≤ 0.010) higher peak/mean contact stress and peak/mean chronic contact stress‐time exposure globally and in the superior‐anterior region when compared with asymptomatic hips (Figure 6).

### 3.2 Relationship Between Acetabular Morphology and Contact Mechanics When Correcting for Acetabular Sphericity

**Figure 7:**
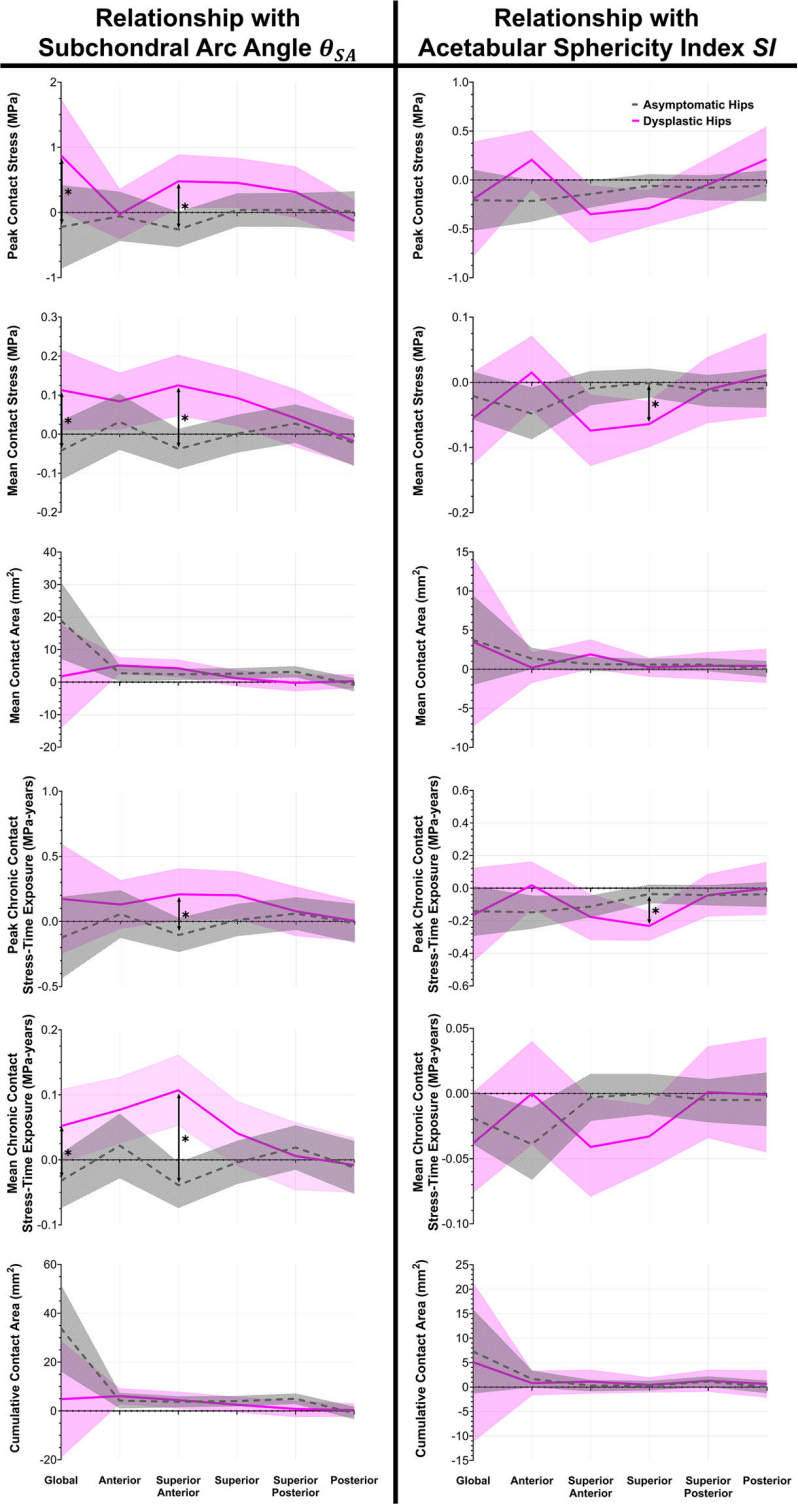
Lines indicate slopes of the mixed‐effect linear models for both asymptomatic (gray dashed) and dysplastic (pink solid) hips. Shaded regions indicate the corresponding 95% confidence interval. * Indicates statistically significant *p* < 0.05 after correcting for multiple comparisons. (Left) Regional relationships between 3D subchondral arc angle *θ*_*SA*_ and contact mechanics metrics (when correcting for acetabular sphericity index *SI*). Dysplastic hips had significantly different superior‐anterior relationships between *θ_SA_* and metrics of peak/mean contact stress and peak/mean chronic contact stress‐time exposure than asymptomatic hips. (Right) Regional relationships between *SI* and contact mechanics metrics (when correcting for *θ_SA_*). Dysplastic hips had significantly different superior relationships between *SI* and metrics of mean contact stress and peak chronic contact stress‐time exposure than asymptomatic hips.

When correcting for acetabular sphericity (SI), the relationship between weight‐bearing acetabular morphology (subchondral arc angle [$\theta_{\mathrm{SA}}$]) and contact mechanics metrics showed markedly different trends between dysplastic and asymptomatic hips in different acetabular regions (Figure 7, left column). In dysplastic hips, increasing global $\theta_{\mathrm{SA}}$ was associated with *increasing* global peak/mean contact stress (*p* ≤ 0.047); this significantly (*p* ≤ 0.047) differed from asymptomatic hips, which demonstrated no significant associations (*p* ≥ 0.228). Similarly, increasing superior‐anterior $\theta_{\mathrm{SA}}$ in dysplastic hips was associated with *increasing* superior‐anterior peak/mean contact stress and peak/mean chronic contact stress‐time exposure (*p* ≤ 0.039). When correcting for multiple comparisons, these associations were significantly (*p* ≤ 0.009) different from those of asymptomatic hips, in which increasing superior‐anterior $\theta_{\mathrm{SA}}$ was associated with *decreasing* mean chronic contact stress‐time exposure (*p* = 0.031).

### 3.3 Relationship Between Acetabular Sphericity and Contact Mechanics When Correcting for Acetabular Morphology

When correcting for weight‐bearing acetabular morphology (subchondral arc angle [$\theta_{\mathrm{SA}}$]), dysplastic hips again demonstrated different relationships between acetabular sphericity (SI) and contact mechanics metrics than asymptomatic hips (Figure 7, right column). In dysplastic hips, increased superior SI (i.e., more spherical) was associated with decreased superior mean contact stress and peak chronic contact stress‐time exposure (*p* < 0.001). When correcting for multiple comparisons, these associations were significantly (*p* ≤ 0.003) different from those of asymptomatic hips, in which increased superior SI was not associated with superior mean contact stress (*p* = 0.948) or peak chronic contact stress‐time exposure (*p* = 0.213).

### 3.4 Relationship Between Femoral Head Coverage and Contact Mechanics When Correcting for Joint Congruency

**Figure 8:**
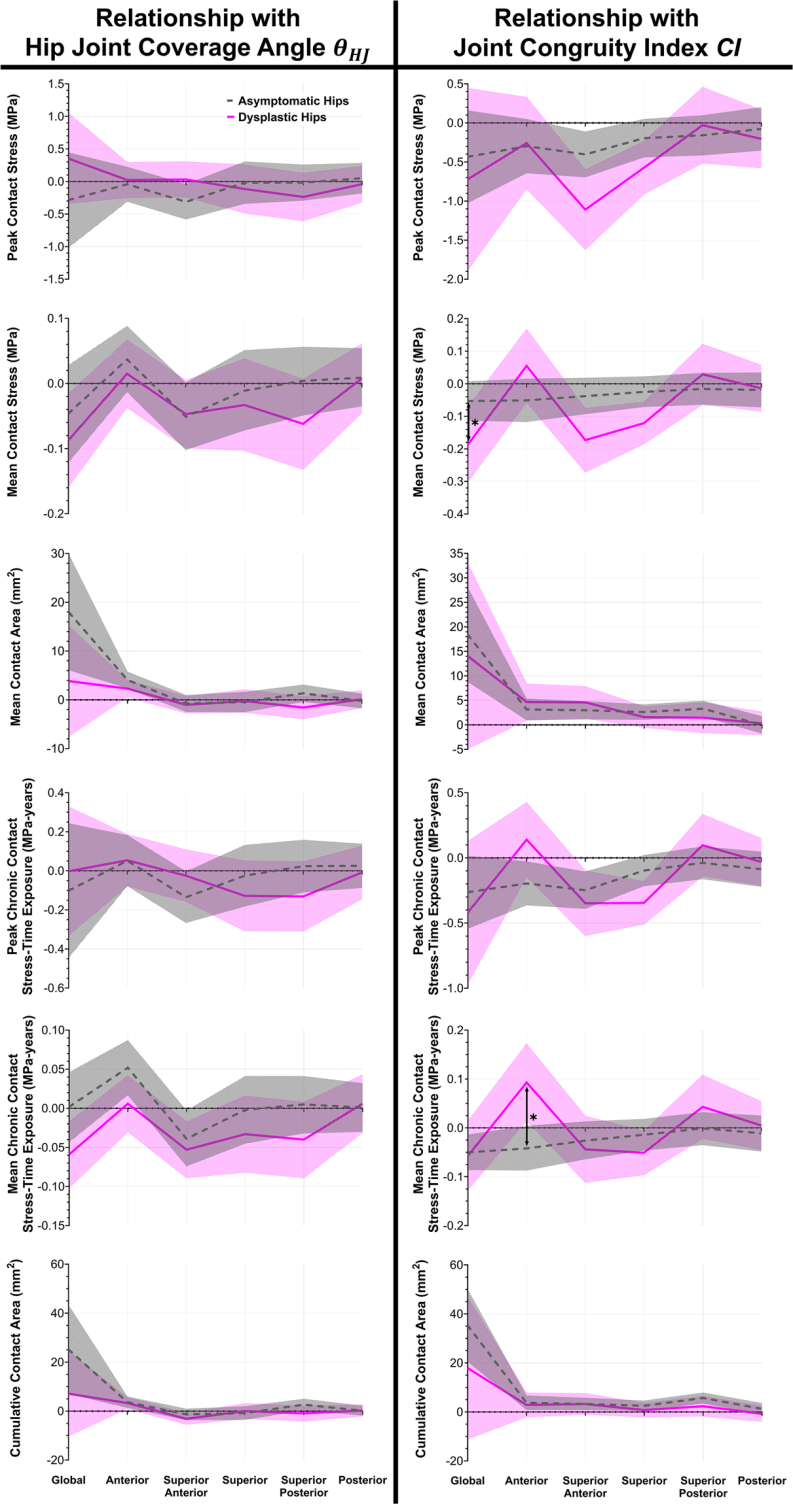
Lines indicate slopes of the mixed‐effect linear models for both asymptomatic (gray dashed) and dysplastic (pink solid) hips. Shaded regions indicate the corresponding 95% confidence interval. * Indicates statistically significant ﻿﻿﻿﻿*p* < 0.05 after correcting for multiple comparisons. (Left) Regional relationships between 3D hip joint coverage angle *θ_HJ_* and contact mechanics metrics (when correcting for joint congruity index *CI*). Asymptomatic and dysplastic hips did not have any significant differences in the relationships between *θ_HJ_* and contact mechanics metrics. (Right) Regional relationships between *CI* and contact mechanics metrics (when correcting for *θ_HJ_*). The relationship between anterior *CI* and anterior mean chronic contact stress‐time exposure was significantly different between asymptomatic and dysplastic hips.

When correcting for joint congruency (joint congruity index [CI]) (Figure 8, left column), asymptomatic and dysplastic hips had similar relationships between coverage of the femoral head by the acetabulum (hip joint coverage angle [$\theta_{\mathrm{HJ}}$]) and contact mechanics metrics (all *p* ≥ 0.051).

### 3.5 Relationship Between Joint Congruency and Contact Mechanics When Accounting for Femoral Head Coverage

When correcting for hip joint coverage angle ($\theta_{\mathrm{HJ}}$), dysplastic hips demonstrated different relationships between joint congruency (CI) and contact mechanics than asymptomatic hips (Figure 8, right column). In dysplastic hips, increasing global CI (i.e., more congruent) was associated with *decreasing* mean contact stress (*p* = 0.008); this significantly (*p* = 0.045) differed from asymptomatic hips, which demonstrated an insignificant association (*p* = 0.076). However, increasing anterior CI in dysplastic hips was associated with *increasing* anterior mean chronic contact stress‐time exposure (*p* = 0.020). When correcting for multiple comparisons, this association was significantly (*p* = 0.003) different than asymptomatic hips, which demonstrated an insignificant (*p* = 0.076) trend of *decreasing* anterior mean chronic contact stress‐time exposure with increasing anterior CI.

## 4 Discussion

The objective of this study was to investigate differences in the relationship between 3D acetabular coverage and contact mechanics for asymptomatic and dysplastic hip joints. Contrary to expectations based on the assumption of a spherical joint shape, a larger span (3D angular arc) of the superior‐anterior acetabular lunate was associated with *increased* superior‐anterior contact stress in dysplastic hips. This was opposite the expected trend of increased lunate arc being associated with *decreasing* contact stress that was found in asymptomatic hips. Increasing spherical shape of the superior acetabulum was significantly associated with decreasing superior contact stress in dysplastic hips but not in asymptomatic hips. As the congruity of the anterior joint increased, there was an unexpected *increase* in anterior chronic contact stress‐time exposure for dysplastic hips, while asymptomatic hips showed the expected (yet insignificant) trend of *decreased* anterior chronic contact stress‐time exposure. These results emphasize fundamental differences in how 3D acetabular coverage and joint congruity affect contact mechanics metrics between asymptomatic and dysplastic hip joints. Asymptomatic hips appear to follow the geometry‐based expectation that greater acetabular coverage of the femoral head will decrease contact stress; however, joint incongruities, particularly acetabular flattening/upturning, prevented this from holding true for dysplastic hips.

Previous work implementing 3D coverage assessments have computed the angular weight‐bearing acetabular arc on 3D bony surface models [11, 12, 13], the percentage of femoral head coverage by projecting the 3D acetabular surface area onto the femoral head surface [14, 15, 16, 17, 18], the curvature mismatch between the acetabulum and femoral head [19], 2D acetabular coverage angles on radially reformatted CT slices at multiple acetabular clockface positions [20, 21, 22], and 3D angles between a horizontal acetabular reference axis and an axis from the femoral head center to the lateral edge of the acetabular rim [23]. However, these 3D techniques differ substantially from how coverage of the femoral head is clinically assessed (i.e., radiographic evaluation of 2D angular coverage relative to a vertical axis), making translation into clinical practice more difficult. Here, we utilize 3D coverage measures that, when measured at the 12 o'clock acetabular position, are essentially the 3D equivalent of the 2D lateral center edge angle (LCEA) and acetabular arc angle measurements performed as clinical coverage assessments [7], providing 3D coverage assessments that more readily relate to how coverage is assessed clinically than previously described techniques. Further, while prior 3D acetabular coverage investigations have demonstrated variable acetabular coverage deformity types and severities in patients with hip dysplasia [12, 20, 22], these studies have not related regional coverage deficiencies to hip contact mechanics. Similarly, in studies of contact mechanics in dysplastic hips that have demonstrated contact stress elevations and reduced contact areas compared to normal hip joints [26, 27], 3D coverage and congruity evaluations were not performed. The present study directly identified significant differences in how hip contact mechanics relate to variations in 3D acetabular coverage and joint congruity.

The significant association between increasing weight‐bearing acetabular surface (subchondral arc angle) and increasing contact stress in dysplastic hips potentially indicates a counterintuitive relationship. However, subchondral arc angle solely measures the angular arc of the acetabular lunate, which is not a measure of acetabular coverage of the femoral head. Therefore, it is possible to have increased acetabular arc length that is shaped (e.g., flattened) or oriented (e.g., upturned laterally) in a way that does not increase functional, weight‐bearing femoral head coverage. This is supported by the negative association observed between acetabular sphericity index (greater sphericity index indicates more spherical acetabulum) and contact stress in dysplastic hips. In cases where the anterior acetabular shape is more flattened or oriented such that it is not in contact with the femoral head, the anterior acetabulum may not experience loading; however, in cases with more spherical acetabular shapes, the anterior region may support increased loading and thereby experience higher contact stress. In cases with acetabular flattening or upturned acetabula, use of radiographic measures beyond the standard LCEA, such as the Tönnis angle (acetabular index), may better estimate hip joint congruity and contact mechanics.

The hip joint coverage angle did not have a significant association with any contact mechanics metric in either asymptomatic or dysplastic hips. This angle measures lateral acetabular coverage over the femoral head, and in the superior region, it is essentially the 3D equivalent of the 2D LCEA. However, previous work has found that acetabular corrections based on normalizing 2D radiographic measures of coverage like LCEA do not guarantee optimal, or even improved, contact mechanics [5, 6, 26, 28]. The current study similarly suggests that like common 2D measures, 3D equivalent measures of femoral head coverage alone may not sufficiently describe contact mechanics.

For the asymptomatic subjects modeled in this study, medical records were screened for diagnoses of congenital or developmental hip pathology, neuromuscular diagnoses, and indications of hip pain. However, we cannot completely rule out the potential of these subjects having undiagnosed hip pathology, as not all subjects with radiographic abnormalities have hip pain or seek orthopedic treatment. While radiographic measurements were used to assess 2D acetabular coverage for comparison to the cohort of dysplastic hips, they were not used to exclude subjects from inclusion in the study due to undiagnosed hip deformity. Due to this radiographic evaluation, we identified 6/50 (12%) asymptomatic subjects with LCEA < 20° and 2/50 (4%) as having LCEA > 40°. These proportions agree with prior studies investigating radiographic findings of acetabular under‐ or over‐coverage in the general adult population [40, 41], and excluding asymptomatic subjects with LCEA < 20° did not impact the findings of this study. Still, caution should be used when interpreting the findings of this cohort as it is more representative of asymptomatic hips rather than radiographically “normal” hip joints.

There are several limitations to this study regarding the 3D coverage calculations. Patient‐specific hip models were obtained from segmentations of supine CT scans/angiograms. While these hip models were reoriented to an upright, neutral orientation, the femoral head position relative to the acetabulum during supine CT imaging was maintained. There is a possibility the combined effects of hip laxity and gravity on the position of the femoral head within the acetabulum, as well as defining the femoral head center using a best‐fit sphere, could have subtly influenced the computed 3D coverage measures. In selected cases, including the cam deformity in the femoral head ROI definition resulted in an approximate 0.1% change in the computed subchondral arc angles and 0.2% change in the hip joint coverage angles, differences which are unlikely to significantly impact the study findings. Furthermore, this study only estimated bony 3D coverage based on CT imaging, which excludes coverage provided by the acetabular labrum and potentially results in underprediction of functional coverage. Additionally, factors such as supine/standing orientation and pelvic tilt are known to affect functional femoral head coverage [42, 43], highlighting the importance of patient‐specific orientation on 3D coverage calculations. Given that hip joint weight‐bearing CT is only in its infancy [44, 45], supine CT scan acquisition remains the universal standard for clinical assessment of coverage and was used for this study. However, this prevented inclusion of patient‐specific postural changes to increase hip stability in the computed 3D coverage calculations. Future studies should investigate how patient‐specific functional orientation influences the femoral head position relative to the acetabulum and the computed 3D coverage angles.

There are also several limitations related to the DEA modeling. First, DEA is a rapidly executing, highly numerically stable computational modeling technique, which requires several inherent simplifications, such as the omission of labrum mechanics and assumption of rigid underlying bone [46, 47]. However, contact stresses computed using this DEA methodology have been validated against contact pressures measured directly in cadaveric hip joints and have been found to be accurate [30, 34]. The use of literature‐based average walking gait loading patterns is another limitation of this study. We have previously shown that contact stresses computed with our DEA methodology are sensitive to the applied loading scheme [48], and dysplastic coverage morphology variations have been found to affect hip joint reaction forces [49]. Given that patient‐specific gait information was not available for the subjects in this study, loading the subject‐specific DEA models with average gait data obtained from individuals that most closely match the subject populations in this study (i.e., dysplastic gait for dysplastic DEA models and gait from matched controls for asymptomatic DEA models) provided the most accurate gait representation possible for contact mechanics assessment. Finally, the CT imaging used to generate the hip models in this study did not include the knee, necessitating surface matching of each patient‐specific femoral model to a full‐length template femur model for model orientation. This template‐based approach has been shown to insignificantly influence DEA‐computed contact mechanics when modeling literature‐based gait [32]. Nevertheless, current clinical practice for evaluating hip dysplasia involves femoral version assessment on 3D CT or MRI imaging [50], and our DEA modeling technique incorporates patient‐specific femoral version whenever possible [32].

Defining the relationship between 3D acetabular coverage and hip contact mechanics may provide surgeons information regarding how to better reduce abnormal contact stresses without the need for full patient‐specific computational models. In this study, fundamental differences in how 3D acetabular coverage and joint congruity affect contact mechanics were identified between asymptomatic and dysplastic hip joints. As such, dysplastic hips should not be expected to respond mechanically the same to changes in coverage as do asymptomatic hip joints.

## Author Contributions

H.D.A. performed computational modeling, assisted with study design, performed radiographic/DRR evaluation, wrote and approved the manuscript. J.E.G. assisted with study design, assisted with computational modeling, wrote and approved the manuscript. W.M.S. performed computational modeling, performed radiographic/DRR evaluation, wrote and approved the manuscript. D.J.L.R. assisted with computational modeling, performed radiographic/DRR evaluation, wrote and approved the manuscript. K.P. performed statistical analysis, wrote and approved the manuscript. N.A.G. assisted with statistical analysis, wrote and approved the manuscript. M.C.W. performed radiographic evaluation, wrote and approved the manuscript. J.B.H. designed the study, wrote and approved the manuscript.
